# Triolein alleviates ischemic stroke brain injury by regulating autophagy and inflammation through the AKT/mTOR signaling pathway

**DOI:** 10.1186/s10020-024-00995-5

**Published:** 2024-12-06

**Authors:** Chaoqun Wang, Yuntao Li, Yonggang Zhang, Daniel Smerin, Lijuan Gu, Shuting Jiang, Xiaoxing Xiong

**Affiliations:** 1 Department of Neurosurgery, Renmin Hospital of Wuhan University, Wuhan, China; 2 Central Laboratory, Renmin Hospital of Wuhan University, Wuhan, China; 3 Department of Breast Surgery, Changzhou Maternal and Child Health Care Hospital, Changzhou Medical Center, Nanjing Medical University, Changzhou, China; 4 Department of Anesthesiology, Renmin Hospital of Wuhan University, Wuhan, China; 5 South Texas Research Facility, San Antonio, TX USA

**Keywords:** Autophagy, Inflammation, Neuroprotection, AKT/ mTOR signaling pathway, Ischemic stroke

## Abstract

**Background:**

Triolein, a symmetric triglyceride exhibiting anti-inflammatory and antioxidant properties, has demonstrated potential in mitigating cellular damage. However, its therapeutic efficacy in ischemic stroke (IS) and underlying molecular mechanisms remain elusive. Given the critical roles of inflammation and autophagy in IS pathogenesis, this study aimed to elucidate the effects of triolein in IS and investigate its mechanism of action.

**Methods:**

We evaluated the impact of triolein using both in vitro oxygen-glucose deprivation/reoxygenation (OGD/R) and in vivo middle cerebral artery occlusion (MCAO/R) models. Neurological function and cerebral infarct volume were assessed 72 h post-reperfusion. Autophagy was quantified through monodansyl cadaverine (MDC) labeling of autophagic vesicles and Western blot analysis of autophagy-related proteins. Microglial activation was visualized via immunofluorescence, while inflammatory cytokine expression was quantified using RT-qPCR. The cytoprotective effect of triolein on OGD/R-induced HT22 cells was evaluated using Cell Counting Kit-8 and lactate dehydrogenase release assays. The involvement of the Protein kinase B/Mechanistic target of rapamycin kinase (AKT/mTOR) pathway was assessed through Western blot analysis.

**Results:**

Triolein administration significantly reduced infarct volume, enhanced neurological recovery, and attenuated M1 microglial activation and inflammation in MCAO/R-induced mice. Western blot analysis and MDC labeling revealed that triolein exerted an inhibitory effect on post-IS autophagy. Notably, in the BV2-induced OGD/R model, triolein demonstrated an autophagy-dependent suppression of the inflammatory response. Furthermore, triolein inhibited the activation of the AKT/mTOR signaling pathway, consequently attenuating autophagy and mitigating the post-IS inflammatory response.

**Conclusions:**

This study provides novel evidence that triolein exerts neuroprotective effects by inhibiting post-stroke inflammation through an autophagy-dependent mechanism. Moreover, the modulation of the AKT/mTOR signaling pathway appears to be integral to the neuroprotective efficacy of triolein. These findings elucidate potential therapeutic strategies for IS management and warrant further investigation.

**Graphical abstract:**

**Figure:**
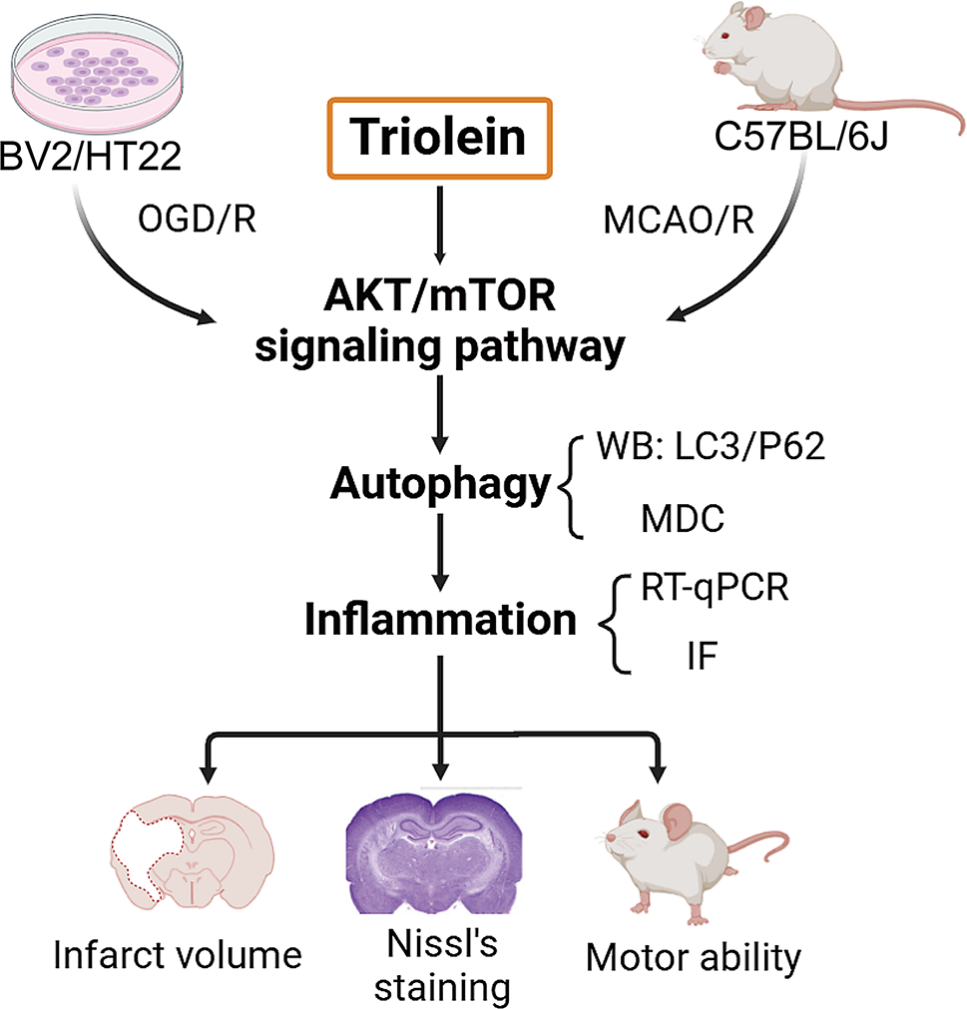
Graphical abstract was created with BioRender.com

**Supplementary Information:**

The online version contains supplementary material available at 10.1186/s10020-024-00995-5.

## Introduction

Stroke is the second leading cause of death globally, with ischemic stroke (IS) constituting approximately 87% of all stroke cases (Ajoolabady et al. 2021). Currently, primary therapeutic modalities for IS encompass pharmaceutical thrombolysis and mechanical thrombectomy (Mendelson and Prabhakaran 2021). Although significant advances have been made in treatments targeting post-stroke hemodialysis, these interventions have failed to comprehensively address secondary cellular damage after stroke resulting in brain injury (Ren et al. 2020). There is a pressing need to investigate more potent therapeutic approaches to address this issue.

Inflammation plays a pivotal role in the development of ischemic injury (Endres et al. 2022). In the early stages of stroke, the infarct area releases a substantial amount of matrix metalloproteinases (MMPs) and pro-inflammatory cytokines. Subsequently, microglia in the brain respond to the damage, aid in the clearance of debris from necrotic cells, and contribute to neural circuit repair; while exhibiting distinct phenotypes and functions during ischemic brain injury (Peng et al. 2022). The inflammation mediated by the classical activated M1 phenotype is a crucial factor leading to neuronal death and expansion of the infarcted area. The inflammatory cytokines induced by this phenotype, including tumor necrosis factor-alpha (TNF-α), interleukin-1β (IL-1β), and interleukin-6 (IL-6), prolong the inflammatory response and exacerbate brain damage following stroke (Zeng et al. 2022; Xue et al. 2023). Hence, modulating the polarization of microglia towards the M1 phenotype is crucial for mitigating inflammatory damage and improving neurological outcomes after stroke.

Autophagy, also recognized as type II programmed cell death, is a metabolic process involving cell death and waste recycling (Maiuri et al. 2007). In IS, the process of autophagy remains a double-edged sword, with its role still being a subject of debate. Autophagy is crucial in maintaining cellular homeostasis and biological survival during ischemic injury (Rao et al. 2020). However, excessive or prolonged autophagy activation can be detrimental to neurons or other cells. Numerous studies have reported that stroke can induce an enhanced autophagic response, triggering neural damage, including neuronal apoptosis and blood-brain barrier disruption (Kim et al. 2020). Autophagy activation mediated by the AKT/mTOR signaling pathway and regulated by reactive oxygen species (ROS) may contribute to microglial apoptosis in IS brain injury (Chen et al. 2016). At the molecular level, autophagy is mainly executed by multiple autophagy-related genes (Atg) and is regulated by a complex signaling network. Recent investigations have shown that AKT or Protein Kinase C (PKC) can regulate mTOR-mediated autophagy in diverse cell types (Choi et al. 2013). However, whether triolein can modulate autophagy through the AKT/mTOR pathway remains unclear in IS.

Numerous investigations have discovered a bidirectional interaction between inflammation and autophagy. For instance, autophagy can trigger the release of several inflammatory components and accelerate the inflammatory process by activating inflammasomes like NOD-like receptor thermal protein domain associated protein 3 (NLRP3). Conversely, cytokine production during inflammation can impact autophagy (Espinosa-Garcia et al. 2020). Interferon-γ, TNF-α, IL-1, IL-2, IL-6, and transforming growth factor-β have been demonstrated to induce autophagy (Qian et al. 2017).

Triolein, a symmetric triglyceride, demonstrates robust anti-inflammatory and antioxidant properties. It can hinder MMP-1 up-regulation and reduce ROS and IL-6 expression in irradiated keratinocytes (Leirós et al. 2017). However, the therapeutic effect of triolein in IS and its underlying mechanism remain unknown.

This study investigated the neuroprotective impact of triolein administration in vivo and in vitro. Specifically, we examined how triolein affects autophagy and inflammation after an IS. Additionally, we explored the involvement of the AKT/mTOR signaling pathway in the neuroprotective role of triolein in IS. These data will help to understand the effects of triolein on IS as well as its underlying mechanism, and provide insights into the clinical treatment of IS.

## Materials and methods

### Mice

One week before the experiment, adult male C57BL/6J wild-type mice (weighing 25-30 g, aged 8-10 weeks) were procured from the Wuhan University Center for Animal Experiments. The mice were housed at the Renmin Hospital of Wuhan University under controlled conditions: 22 ± 2 ℃ temperature, 65% ± 5% humidity, a 12 h light/dark cycle, and ad libitum access to food and water. Animal protocols were approved by the Wuhan University Animal Care and Use Committee Guidelines (No.WDRM-20240201 A; 2024.2.2——2024.12.9). Edaravone (Eda, 6 mg/kg) was used as a positive control. Using a computer-generated block randomization list without stratification, mice were randomly allocated to one of eight groups: Sham, MCAO/R, MCAO/R + Vehicle, MCAO/R + Triolein-10, MCAO/R + Triolein-20, MCAO/R + Triolein-40, MCAO/R + Triolein-80, and a drug positive control group MCAO/R + Eda.

### Reagents and drug administration

Triolein, bafilomycin A1 (BafA1), and GSK690693 (GSK) were purchased from MedChemExpress (United States). The monodansyl cadaverine (MDC) Assay and lactate dehydrogenase (LDH) Cytotoxicity Assay kits were purchased from Beyotime Biotechnology (China). The concentrations of BafA1 (Kim et al. 2022; Sho et al. 2017) and GSK690693 (Wei et al. 2024; Wang et al. 2022a) were selected based on previously published studies.

In the in vivo experiments, triolein was dissolved in 3% dimethyl sulfoxide (DMSO) and diluted with distilled water (dH_2_O) to achieve the desired concentrations.

Based on the literature, in the first experiment, mice were treated 30 min after MCAO/R with 4 different doses of triolein (10, 20, 40, and 80 mg/kg) by oral gavage twice daily for three days (Shimotoyodome et al. 2011). Mice treated with 40 mg/kg triolein showed the strongest reduction in cerebral infarct volume and anti-inflammatory activity. Therefore, a dose of 40 mg/kg was used in the following studies **(Supplementary S1)**.

In the initial phase of the in vitro experiments, HT22 neurons and BV2 cells were categorized into five groups: (1) a control group incubated at 37 ℃ in a standard CO_2_ (5%) incubator with the same glucose-containing buffer throughout the experiment; (2) an oxygen-glucose deprivation/reoxygenation (OGD/R) group; (3) an OGD/R + Vehicle group; (4) an OGD/R + Triolein group with different concentrations (20, 40, 60, and 80 mM). Subsequent experiments focused on the 40 mM concentration, which effectively reduced the expression of pro-inflammatory cytokines **(Supplementary S3)**; (5) Deiprone (DFP, 125 mg/kg) and 3-n-butylphthalide (NBP, 10 µM) were used as positive controls for BV2 cells and HT22 cells, respectively (Liao et al. 2023; Zhang et al. 2024).

### MCAO/R models

The MCAO/R model was established following the previously described method (Zhang et al. 2023a, b). Throughout the procedure, a thermostatic heating pad was used to maintain the rectal temperature of each mouse at 36.5 ± 0.5 ℃. The unilateral MCAO/R on the left side was induced by employing a 6.0 mm silk (Doccol, Corp., Redlands, CA, USA) to obstruct the left middle cerebral artery origin. One hour after obstruction, the monofilament was removed to initiate reperfusion. Sham control mice underwent the same surgical technique but without ligation.

### 2,3,5-Triphenyl tetrazolium chloride (TTC) staining and infarct volume measurement

The methods adhered to the established protocols (Gu et al. 2014). Specifically, 3 days post-MCAO/R initiation, animals from each group were euthanized for brain infarction volume quantification. Brains were extracted, sectioned into 2 mm coronal pieces using a brain matrix, and immersed in a 2% TTC solution (Solarbio, China) at 37 ℃ for 10 min, followed by fixation in 4% paraformaldehyde for 24 h. ImageJ software was used to calculate the areas of the infarct, ipsilateral hemisphere, and contralateral hemisphere. The infarct volume was determined using the formula: Infarct volume = contralateral hemisphere region ˗ non-infarcted region in the ipsilateral hemisphere. The infarct percentage was calculated as follows: Infarct percentage = infarct volume/volume of the contralateral hemisphere × 100%.

### Ischemic core and penumbra segmentation

The separation of the ischemic core and penumbra was performed as previously described (Yingze et al. 2022). Initially, the brain tissue was extracted following decapitation and placed in a mouse brain slicer matrix. At the coronal plane, 3 mm posterior to the frontal pole, the brain tissue was sectioned into three slices with thicknesses of 3 mm (Sect. 1), 4 mm (Sect. 2), and 3 mm (Sect. 3), respectively. Subsequently, Sect. 2 was isolated, and the midline between the ipsilateral and contralateral hemispheres was identified. A sagittal incision was then made 1.5 mm lateral to the midline, followed by a transverse diagonal cut at approximately the “1 o’clock” position. Consequently, the tissue external to the “1 o’clock” direction was designated as the infarct core region, while the cortical tissue between the sagittal incision and the “1 o’clock” direction was identified as the ischemic penumbra (Supplementary Material 6).

### Behavioral tests

Behavioral testing was conducted 3 days post-MCAO/R, preceded by a 1 h acclimation period of mice in the test chamber. The Logan score (Zhu et al. 2022a, b) was selected as the reference standard and categorized as follows: 0 = no deficit; 1 = forelimb weakness and body turning to the ipsilateral side when held by the tail; 2 = circling to the ipsilateral side; 3 = unable to support weight on the affected side; 4 = no spontaneous activity or barrel rolling.

As previously reported, neurological deficits were assessed using the 9-point scale (Li et al. 2022) (0–8 scale) as follows: 0 = no neurological deficits; 1 = left forelimb flexion upon suspension by the tail or failure to fully extend the right forepaw; 2 = left shoulder adduction upon suspension by the tail; 3 = reduced resistance to a lateral push toward the left; 4 = spontaneous movement in all directions with circling to the left only if pulled by the tail; 5 = circling or walking spontaneously only to the left; 6 = walking only when stimulated; 7 = no response to stimulation; 8 = stroke-related death.

The Bederson score (Liu et al. 2012; Bieber et al. 2019) was used to determine the neurological deficits on a 0–5 scale as: 0 = no motor deficits; 1 = forelimb flexion without other abnormalities; 2 = reduced resistance to forelimb flexion and lateral push toward the paralyzed side; 3 = exhibited circling behavior; 4 = appearance of longitudinal rotation; 5 = no movement.

As previously reported, Hanging score (Patel et al. 2023) was evaluated using the following 0–5 scale: 0 = fall off the wire; 1 = hanging with forelimbs; 2 = hanging with forelimbs and attempting to climb up; 3 = hindlimbs involved in hanging; 4 = hanging with four limbs and tail; 5 = successful escape.

### Immunofluorescence staining

Brain tissues were sectioned into 50 μm coronal slices following paraffin immersion and preservation in 4% paraformaldehyde (Gu et al. 2012). Slices were blocked for one hour using a solution comprising 0.1 M phosphate buffer saline (PBS), 5% fetal bovine serum (FBS), and 0.3% Triton X. After three 10 min PBS washes, brain sections were incubated overnight at 4 ℃ in a humidified environment with primary antibodies, including anti-CD86 (1:200; Cell Signaling Technology) and anti-Iba1 (1:200; Cell Signaling Technology). Following primary antibody incubation, samples were treated with Alexa-488 (green, Invitrogen) or Alexa-594 (red, Invitrogen) -conjugated antibodies. Diamidino-2-phenylindole (DAPI) stain was applied to the sections, and the total number of Iba1-positive and CD86-positive microglia in the ischemic penumbra were quantified in five different fields of view for each section by an observer blinded to the treatment group using a fluorescence microscope (Olympus, Tokyo, Japan). The ImageJ program facilitated the counting of target cell numbers.

### Real-time quantitative PCR (RT-qPCR)

Following the manufacturer’s guidelines, total RNA was extracted from the homogenized brain tissue or cell pellet using TRIzol reagent (Agbio, China). Subsequently, mRNA was reverse transcribed to cDNA using a cDNA synthesis kit (TaKaRa, Shiga, Japan). A mixture of 1 µL of synthesized cDNA and specific primers (Table 1) was used to amplify inflammatory cytokine target genes with SYBR Premix Ex Taq2 (TaKaRa, Shiga, Japan). The RT-qPCR cycling protocol involved an initial step at 50 ℃ for 2 min, followed by 95 ℃ for 10 min, and 45 cycles of 95 ℃ for 10 s, 60 ℃ for 10 s, and 72 ℃ for 15 s. Glyceraldehyde-3-phosphate dehydrogenase (GAPDH) was utilized as a housekeeping and internal control gene to assess the relative expression of the target genes.

**Table 1:**
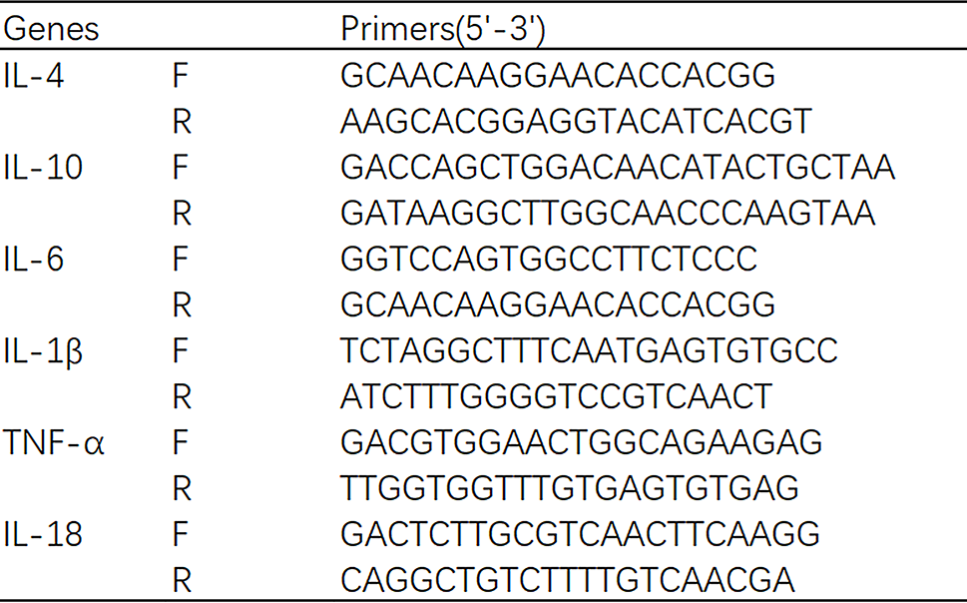
Primers for RT qPCR

### OGD/R model

Mouse hippocampal (HT22, Center for Type Culture Collection, Hubei, China) and murine microglia (BV2, Center for Type Culture Collection, Hubei, China) cell lines were cultured in Dulbecco’s modified Eagle’s medium (DMEM, Procell Life Science & Technology Co., Ltd, Zhejiang, China) supplemented with 10% FBS (Procell Life Science & Technology Co., Ltd, Zhejiang, China) in a 5% CO_2_ incubator. Upon reaching the logarithmic phase, cells were washed twice with PBS and exposed to glucose-free DMEM to simulate OGD injury. The cells were then subjected to hypoxic conditions (1% O_2_, 5% CO_2_, and 94% N_2_) for a specified duration at 37 ℃ in a hypoxic incubator (Binder, CB-210 hypoxia workstation). Subsequently, glucose was reintroduced in DMEM, and the cultures were allowed to recover for 12 h under normoxic conditions (37 ℃, 5% CO_2_), as previously described (Xiong et al. 2014). Our investigation revealed that approximately 50% of the cells died after 6 h of OGD induction **(Supplementary S2)**. Consequently, subsequent experimental conditions were established for 6 h. Control groups, devoid of OGD exposure, were washed twice with PBS, remained in DMEM, and were not subjected to oxygen deprivation.

### Western blots

The Western blotting procedure was performed as previously described (Wang et al. 2022a, b). Total protein extraction was conducted from HT22 or BV2 cells subjected to various conditions, and ipsilateral brain tissue was collected 3 days post-MCAO/R. Ice-cold RIPA buffer (Applygen, China) supplemented with proteinase and phosphatase inhibitors (Servicebio, China) was used for tissue homogenization and lysis on ice. Cell suspensions were lysed through sonication, and the resulting homogenates were centrifuged at 4 ℃ for 30 min at 12,000 × g to obtain supernatants. Protein content was determined using the BCA kit (Servicebio, China). Electrophoresis on 4–15% sodium dodecyl sulfate-polyacrylamide gels separated protein samples (10 µL/lane), which were then transferred to Polyvinylidene fluoride (PVDF) membranes (Millipore, Billerica, MA, United States). The membranes were immersed in 5% non-fat milk with PBS/0.1% Tween for 2 h, blocked, and subsequently incubated overnight at 4 ℃ with primary antibodies: mouse anti-P62 (Abcam, ab109012, 1:1000), anti-LC3-II/I (Cell Signaling Technology, 12741, 1:1000), anti-mTOR (Affinity, AF6308, 1:1000), pan-AKT1/2/3 (Affinity, AF6261, 1:1000), anti-phosphorylated-mTOR (Affinity, AF3308, 1:1000), and anti-Phospho-AKT1/2/3 (Ser473) (Affinity, AF0016, 1:1,000). Subsequent steps included washing and incubation with appropriate secondary antibodies (Affinity, 1:2000) for 2 h at room temperature. Image assessment was performed using cSeries capture software (Azure Biosystems, Inc., Dublin, CA, USA), and ImageJ was used to evaluate the optical intensity of bands, normalized to actin (Affinity, AF7018, 1:2000). These experiments were repeated three times.

### Cell viability measurement

Cell viability assessment was performed utilizing a Cell Counting Kit-8 (CCK8, Yeason, China) following the manufacturer’s guidelines. 1 × 10^4^ cells/well of HT22 and BV2 cells were harvested and seeded in 96-well plates. The HT22 and BV2 cells underwent a 6 h pretreatment with triolein or 0.1% DMSO. Subsequently, cells were subjected to OGD/R for 6 h and allowed to recover for 24 h. After establishing the OGD/R model, 10 µL of CCK8 reagent was added to each well and incubated for an hour. Subsequently, absorbance was measured at a wavelength of 450 nm using a microplate reader (Bio-Rad Laboratories, Hercules, CA, USA).

### LDH release assay

Cellular damage was assessed using the LDH Cytotoxicity Assay Kit (Beyotime Biotechnology, China), strictly following the manufacturer’s guidelines. HT22 cells were seeded in 96-well plates (1 × 10^4^ cells/well). Following a 6 h pretreatment with triolein or 0.1% DMSO, HT22 cells underwent a 6 h OGD period, succeeded by 24 h of reperfusion. A 10 µL medium solution was added to each neuronal culture well in a 24-well plate within an optically clear 96-well plate. Subsequently, 100 µL of LDH Reaction Mix was added to each well, thoroughly mixed, and incubated for 30 min at room temperature. Finally, the absorbance was measured at a 490 nm wavelength using a microplate reader (Bio-Rad Laboratories, Hercules, CA, USA).

### MDC assay

The presence of autophagic vacuoles was detected through MDC staining (Peng et al. 2021). After completing all experiments, cells were exposed to MDC at 37 ℃ for 30 min. Following three washes with assay buffer, cells were examined and imaged under a fluorescent microscope (BX51; Olympus, Tokyo, Japan). The MDC fluorescence intensity was quantified as integrated density, representing the sum of pixel values in cells from three distinct images utilizing ImageJ software (NIH, Bethesda, MD, USA).

### Nissl staining

Nissl staining was used to observe the morphological changes of neurons in the ischemic penumbra after 24 h of reperfusion (*n* = 8). Experimental procedures were carried out in strict accordance with the instructions of the Nissl staining kit (#G1430, Solarbio, China).

### Statistical analyses

Statistical analyses were performed using GraphPad Prism 9.0 (GraphPad Software, Inc., San Diego, CA, USA). All values are presented as mean ± standard deviation. Multiple comparisons were conducted utilizing Tukey’s post hoc test following a one-way ANOVA. A *P* < 0.05 was considered statistically significant.

## Results

### Triolein protects against ischemia-induced brain damage in mice

**Fig. 1:**
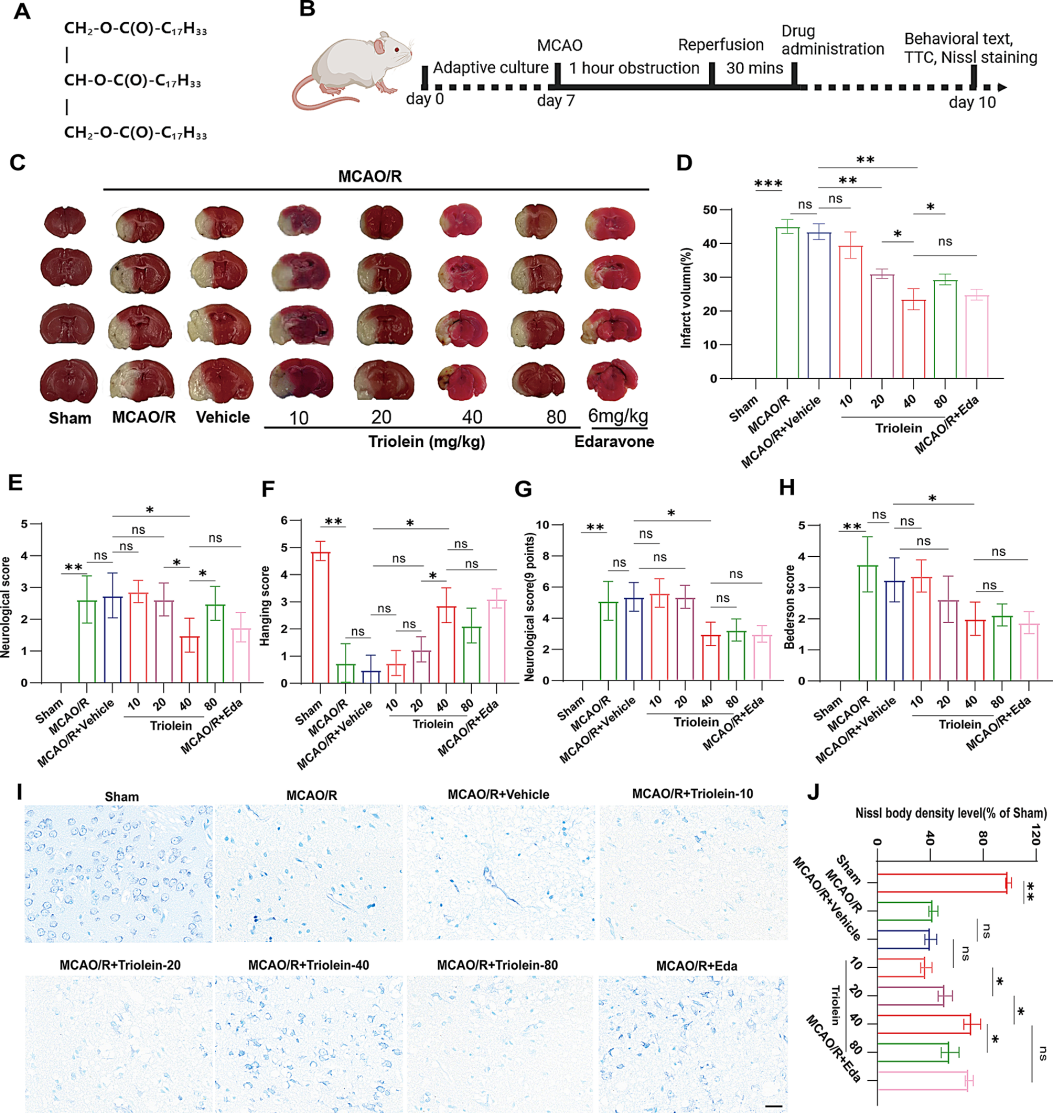
Triolein treatment significantly attenuated cerebral ischemic injury. **A** Molecular formula of Triolein. **B** Flow chart of animal experiments. **C**-**D** Representative TTC staining assay was performed to evaluate the infarct volume and quantified using ImageJ software. **E**-**H** The Longa score, Hanging Test, 9 points scale, and Bederson score were determined 3 days after MCAO/R to evaluate neurological function. I-J Nissl staining showing morphological neuronal changes in the ischemic penumbra 3 days after reperfusion. Scale bars = 20 μm. *n* = 8 per group, *p < 0.05, **p < 0.01, ***p < 0.001 versus the specified group. In addition, there was no statistically significant difference between the MCAO/R group and the MCAO/R + Vehicle group, indicating that the drug solvent had no additional effect on the experimental results

To evaluate the neuroprotective effects of triolein on IS induced brain injury, we employed a comprehensive approach utilizing TTC staining, neurological scoring, and Nissl staining. C57BL/6J mice underwent MCAO/R surgery, followed by triolein administration for three consecutive days. On day 10 post-surgery, we assessed behavioral scores, cerebral infarction volume, and neuronal morphology. The molecular structure of triolein and the experimental timeline are illustrated in Fig. 1A and B, respectively. TTC-stained brain sections (Fig. 1C-D) revealed significant infarction in the MCAO/R model group compared to the sham group, confirming successful establishment of the cerebral ischemia-reperfusion model. The vehicle group showed no significant effect on cerebral ischemic injury compared to the MCAO/R group. Triolein administration at various concentrations reduced the infarct area, with the 40 mg/kg dose demonstrating optimal efficacy in mitigating cerebral ischemia-induced brain tissue infarction. Notably, there was no significant difference in infarct volume reduction between the 40 mg/kg triolein group and the positive control group treated with edaravone (Eda). Neurological function was evaluated on day 10 using a battery of behavioral tests, including the Longa score, Hanging test, 9-point scale, and Bederson score (Fig. 1E-H). The MCAO/R mice exhibited severe neurological deficits compared to the sham group. Triolein treatment significantly improved neurological function, with efficacy comparable to the positive control, edaravone.

Nissl staining revealed morphological alterations in neurons within the ischemic penumbra following cerebral ischemia-reperfusion (Fig. 1I-J). Sham group neurons displayed normal morphology with orderly arrangement and abundant Nissl bodies. In contrast, damaged neurons exhibited cellular atrophy, nuclear shrinkage and pyknosis, disorganized cellular arrangement, indistinct contours, and markedly reduced Nissl bodies. Both MCAO/R and MCAO/R + Vehicle groups showed a significant decrease in Nissl-positive cell proportion compared to the sham group, with no significant difference between these two groups. The MCAO/R + Triolein-10 group showed no significant changes, while the MCAO/R + Triolein-20 group demonstrated a slight increase in Nissl-positive cell proportion. The MCAO/R + Triolein-40 group exhibited a marked elevation in Nissl-positive cell proportion, differing significantly from the MCAO/R + Vehicle group. The MCAO/R + Triolein-80 group showed a slight, but statistically significant, decrease in Nissl-positive cell proportion compared to the MCAO/R + Triolein-40 group. The positive control group (MCAO/R + Eda) displayed a similar proportion of Nissl-positive cells compared to the MCAO/R + Triolein-40 group, with no statistically significant difference between the two groups.

In conclusion, our findings demonstrate that triolein at a concentration of 40 mg/kg exerts significant neuroprotective effects in the context of cerebral ischemia-reperfusion injury, comparable to the positive control edaravone. This suggests that triolein at this optimal concentration may possess potent neuroprotective properties, warranting further investigation as a potential therapeutic agent for ischemic stroke.

### Triolein significantly influences autophagy and inflammation after cerebral ischemic brain injury

**Fig. 2:**
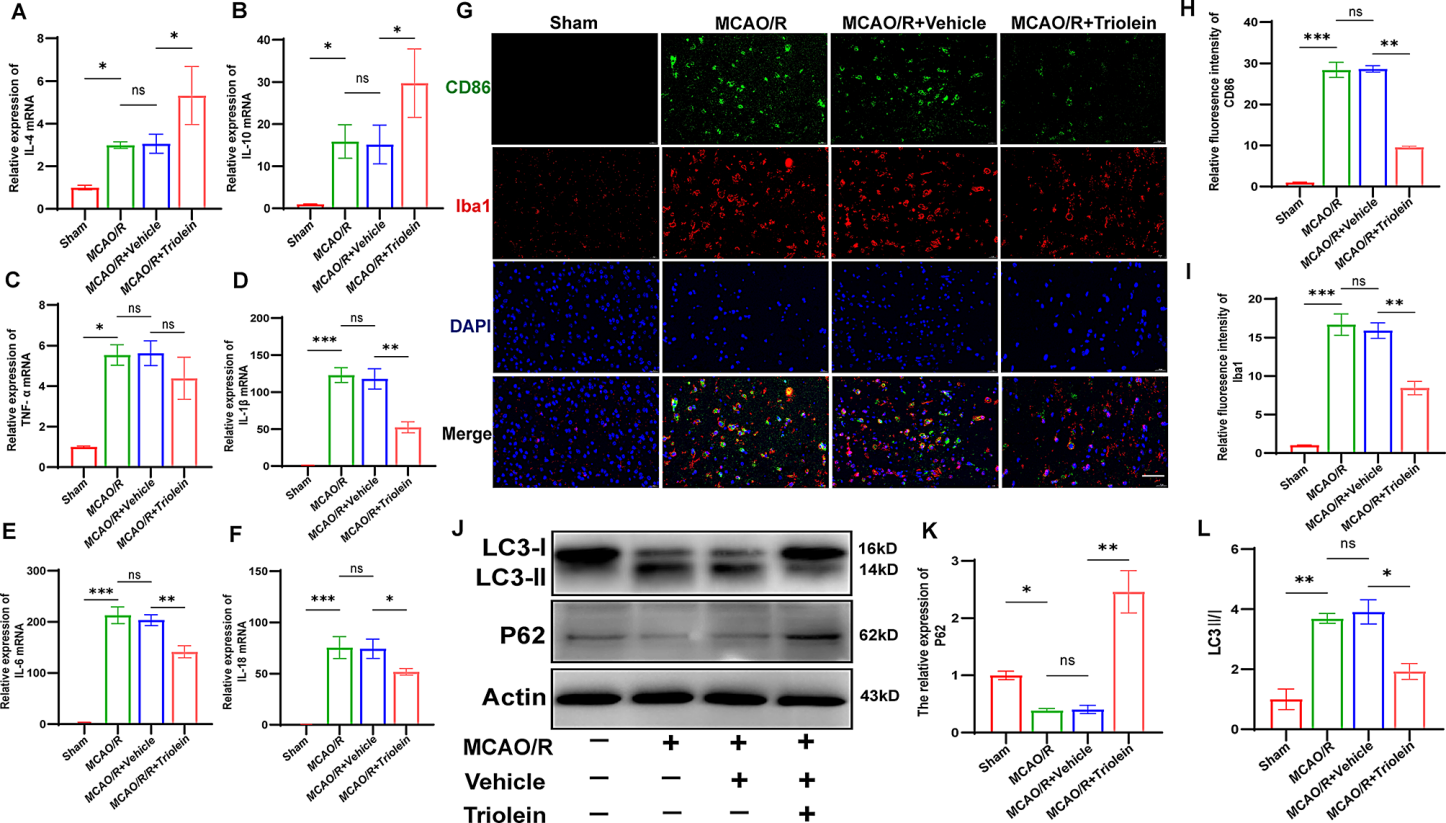
Triolein reduced inflammation and microglial activation and inhibited autophagy after cerebral ischemic injury. **A**–**F** The expression of inflammatory cytokines IL-4, IL-10, TNF-α, IL-1β, IL-6, and IL-18 mRNAs in the ischemic penumbra; GAPDH was used as the control. **G**–**I** The effect of Triolein on activation of proinflammatory microglia after ischemic stroke. Iba1, red, a surface marker of microglia; CD86, green, the surface marker of M1 microglia; DAPI, blue, Nuclei (scale bar, 40 μm). **J**–**L** Western blot to detect the effect of Triolein on LC3-II/I and P62 protein expression in the C57BL/6J ipsilateral cortex after ischemic stroke brain injury. Quantitative results of relative band density were normalized to Actin. *n* = 5 per group, *p < 0.05, **p < 0.01, ***p < 0.001 versus the specified group. In addition, there was no statistically significant difference between the MCAO/R group and the MCAO/R + Vehicle group, indicating that the drug solvent had no additional effect on the experimental results

Upon ischemic stroke onset, copious amounts of pro-inflammatory cytokines are released, amplifying the inflammatory cascade and exacerbating cerebral damage. Simultaneously, microglia transition from a quiescent to an activated state in response to alterations in the external milieu. Numerous studies support the notion that post-stroke brain damage can be reduced by inhibiting microglial activation, particularly by decreasing the polarization of microglia toward the M1 phenotype (Luo et al. 2022). To assess the effect of triolein on the MCAO/R-induced neuroinflammatory response, RT-qPCR was used to detect the IL-4, IL-10, TNF-α, IL-1β, IL-6, and IL-18 mRNA production levels in the ischemic cerebral cortex area. As shown in Fig. 2A–B, triolein administration elevated the IL-4 and IL-10 expression compared to the MCAO/R + Vehicle group. Post-IS, the release of TNF-α, IL-1β, IL-6, and IL-18 mRNAs increased considerably **(**Fig. 2C–F**)**. Triolein therapy significantly suppressed the expression of these pro-inflammatory cytokines.

Subsequently, microglial polarization within the ischemic penumbra was detected 3 days post-MCAO/R. The MCAO/R was found to elevate the numbers of Iba1-positive microglia and CD86-positive pro-inflammatory microglia in the MCAO/R group compared to the Sham group. Conversely, triolein treatment significantly reduced the counts of Iba1-positive microglia and CD86-positive pro-inflammatory microglia **(**Fig. 2G–I**)**. Collectively, these findings suggest that triolein holds the potential to alleviate the inflammatory response and attenuate the polarization of M1 microglia following ischemic stroke.

Moreover, autophagy has been implicated in IS (Yang et al. 2022). To assess the impact of triolein on post-IS autophagy, certain autophagy-related proteins, including microtubule-associated protein light chain 3-I (LC3-I), LC3-II, and P62 were examined in MCAO/R-treated mice. IS enhanced LC3-II/I protein expression and decreased P62 protein expression compared to the Sham group, suggesting that stroke leads to autophagy activation. Triolein treatment notably reduced the LC3-II/I ratio and increased P62 protein expression in the MCAO/R + Triolein group compared to the MCAO/R + Vehicle group **(**Fig. 2J–L**)**. These results signify that triolein holds the potential to mitigate inflammation and autophagy related to cerebral ischemic injury.

### Involvement of the AKT/mTOR signaling pathway in Triolein’s effect on autophagy and inflammation during IS

The AKT/mTOR signaling pathway is among the key mechanisms regulating autophagy and inflammation (Wei et al. 2016; Wang et al. 2020). We investigated the effect of triolein on the AKT/mTOR signaling pathway after IS-induced brain injury. Since autophagy is repressed by the activation of this signaling pathway, we postulated that triolein could potentially activate the AKT/mTOR signaling pathway, thereby suppressing heightened post-stroke autophagy.

**Fig. 3:**
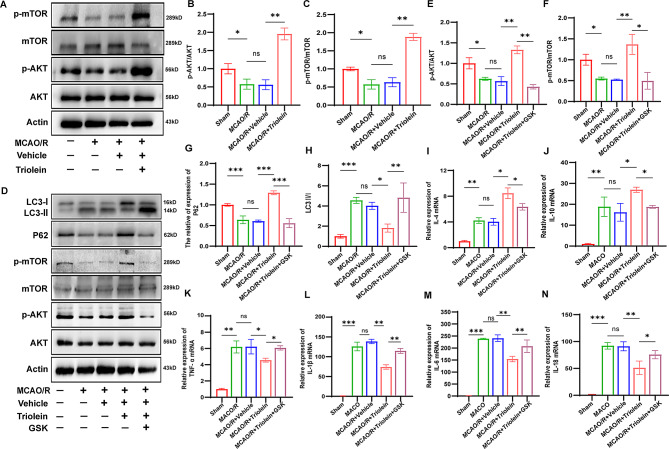
Triolein downregulates autophagy and inflammation via AKT/mTOR signaling. **A**–**C** Representative western blot showing AKT/mTOR expression after MCAO/R and Triolein treatment. Quantitative results of relative band density were normalized to Actin. **D**–**H** Representative western blot of AKT/mTOR signaling pathway and autophagy alterations following GSK-690,693, an autophagy inhibitor, treatment. Quantitative results of relative band density are normalized to Actin. **I**–**N** The expression of the IL-4, IL-10, TNF-α, IL-1β, IL-6, and IL-18 mRNAs in BV2 after GSK-690,693 treatment. GAPDH was used as the control. *p < 0.05, **p < 0.01, ***p < 0.001 versus the specified group, *n* = 3 per group. There was no statistically significant difference between the MCAO/R group and the MCAO/R + Vehicle group, indicating that the drug solvent had no additional effect on the experimental results

As indicated by the decreased ratio of phosphorylated to total AKT and mTOR, AKT/mTOR activity declined in the ischemic group compared to the Sham group. Triolein treatment markedly augmented the phosphorylated levels of AKT and mTOR compared to the MCAO/R + Vehicle group, indicating AKT/mTOR signaling pathway activation by triolein **(**Fig. 3A–C**)**. To validate our hypothesis, we employed GSK-690,693, an ATP-competitive AKT inhibitor, to block the signaling pathway. Comparative analysis (Fig. 3D–F) revealed that the expression levels of p-AKT and p-mTOR proteins in the MCAO/R + Triolein + GSK-690,693 group were diminished compared to the MCAO/R + Triolein group, suggesting successful AKT/mTOR signaling pathway inhibition by GSK-690,693. Following GSK-690,693 administration, the expression level of autophagy-related protein P62 decreased, and the ratio of LC3-II/I increased compared to the MCAO/R + Triolein group (Fig. 3G–H). These findings suggest that the inhibitory effect of triolein on post-stroke autophagy was significantly attenuated after AKT/mTOR signaling pathway blockage, providing preliminary evidence for triolein’s inhibition of post-stroke autophagy through AKT/mTOR signaling pathway activation.

Likewise, the AKT/mTOR signaling pathway is intricately linked to the neuroinflammatory response post-IS. After inhibiting the AKT/mTOR signaling pathway, we examined the expression of inflammatory factors to identify whether triolein’s anti-inflammatory effect is associated with the AKT/mTOR pathway. In the MCAO/R + Triolein + GSK-690,693 group, the mRNA expression of pro-inflammatory cytokines significantly increased, while that of anti-inflammatory cytokines decreased compared to the MCAO/R + Triolein group (Fig. 3I–N). Collectively, these findings indicate that triolein exerts inhibitory effects on post-stroke inflammation and autophagic responses through AKT/mTOR signaling pathway modulation.

### In Vitroevaluation of Triolein’s responsive regulation of autophagy and inflammation

**Fig. 4:**
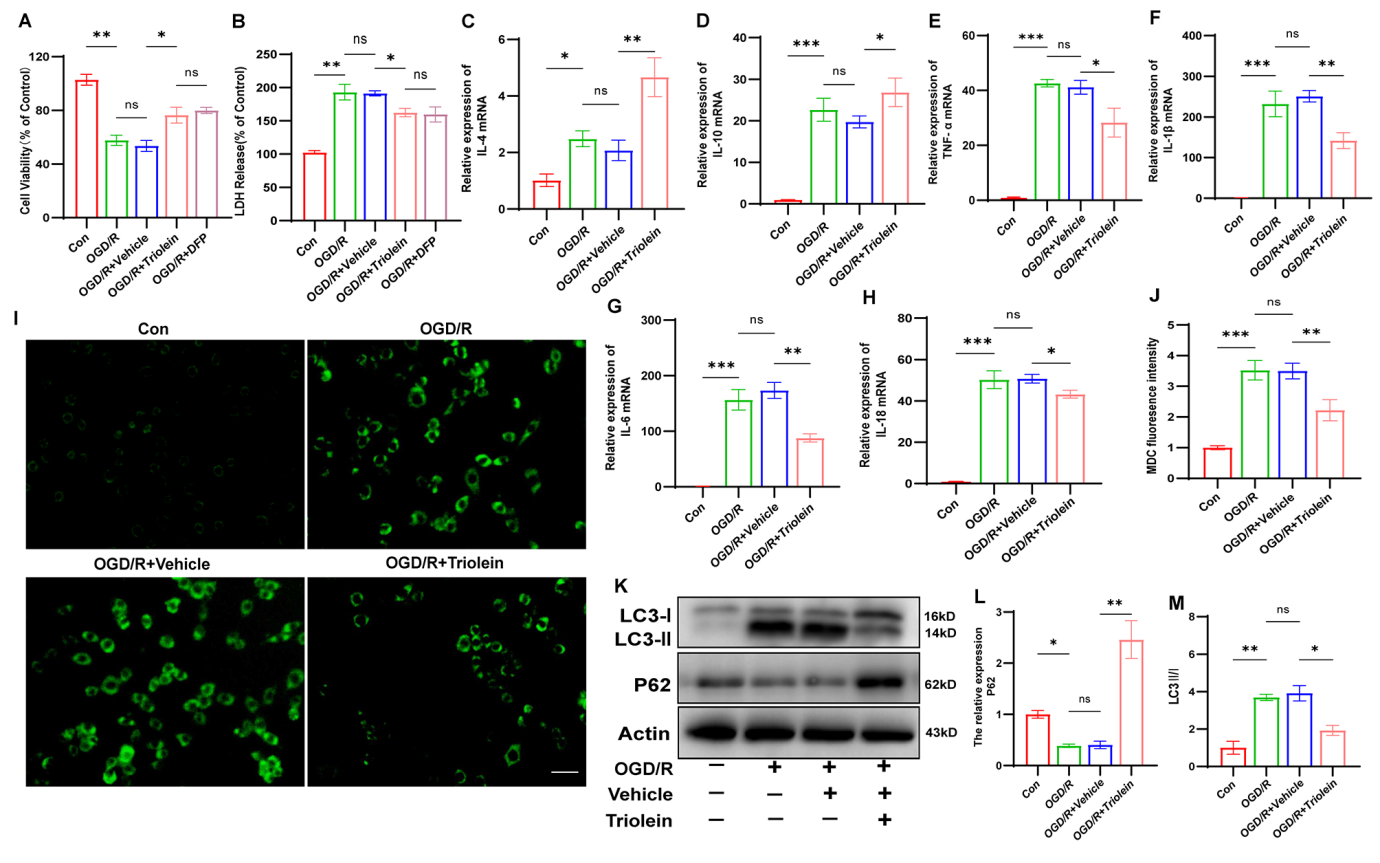
Triolein diminishes autophagic activity and decreases the expression of inflammatory cytokines in OGD/R induced BV2. **A**-**B** CCK8 and LDH release assays demonstrate the protective effect of Triolein against OGD/R in BV2 cells. **C**–**H** The expression of proinflammatory cytokines IL-4, IL-10, TNF-α, IL-1β, IL-6, and IL-18 mRNAs in OGD/R induced BV2 treated with Triolein. GAPDH was used as the control. **I**–**J** MDC labeled autophagic vesicles were used to detect the level of autophagy in OGD/R induced BV2 treated with Triolein (scale bar = 20 μm). **K**–**M** The expression of LC3 and P62 was assessed by western blot in OGD/R induced BV2 treated with Triolein. Quantitative results of relative band density were normalized to Actin. *n* = 3 per group. *p < 0.05, **p < 0.01, ***p < 0.001 versus the specified group. There was no statistically significant difference between the OGD/R group and the OGD/R + Vehicle group, indicating that the drug solvent had no additional effect on the experimental results

The BV2 cells were used in in vitro experiments to investigate the effects of triolein on IS, specifically on autophagy and inflammation. First, we found that Triolein was able to achieve the same protective effect as DFP through the CCK8 and LDH release assay, using the positive drug DFP as a control (Fig. 4A–B). Our findings were consistent with those observed in the in vivo experiments, indicating that the OGD/R + Triolein group exhibited elevated transcription levels of IL-4 and IL-10 compared to the OGD/R + Vehicle group (Fig. 4C–D). Conversely, the OGD/R + vehicle group exhibited a significantly increased expression of TNF-α, IL-1β, IL-6, and IL-18 mRNAs, whereas triolein treatment significantly attenuated the expression of these mRNAs (Fig. 4E–H).

Consistent with our animal experiments, post-OGD/R autophagic activity was assessed by evaluating LC3 and P62 expression. Additionally, autophagic vacuoles, which serve as a surrogate for autophagy levels, were quantified by labeling autophagic vesicles using MDC. Figure 4I–J illustrate a substantial increase in autophagic vacuoles following OGD/R-induced stimulation relative to the control group. Notably, triolein administration significantly reduced OGD/R-induced augmentation of autophagic vacuole levels. Concomitantly, evaluating LC3 and P62 protein levels via Western blot revealed a significant increase in the LC3 II/I ratio with OGD/R compared to the control group (Fig. 4K–M). However, triolein treatment mitigated the OGD/R-induced elevated ratio of LC3-II/I. Similarly, triolein averted the down-regulated expression of P62 caused by OGD/R. These results collectively indicate OGD/R-induced autophagy activation, with triolein demonstrating the ability to counteract post-stroke autophagy activation.

Collectively, our findings suggest that triolein is intricately involved in the neuroinflammatory response and autophagic activity post-IS, aligning with the outcomes observed in in vivo experiments.

### Triolein effectively ameliorates inflammation in an autophagy-dependent manner

In IS, a close link exists between autophagy and inflammation (Cai et al. 2023; Mo et al. 2020). Our preceding animal studies have suggested that triolein’s role in enhancing the inflammatory response post-stroke may be subject to regulation by autophagy. Subsequently, we further investigated the intricate interplay between inflammatory responses and autophagy under triolein intervention in vitro. BafA1, an inhibitor targeting the late stages of autophagy, impedes the fusion of autophagosomes with lysosomes, thereby hindering the acidification and protein degradation within cellular lysosomes (Mauvezin and Neufeld 2015). We used BafA1 in OGD/R-induced BV2 cells at a 100 nM dose based on established literature (Lin et al. 2021).

**Fig. 5:**
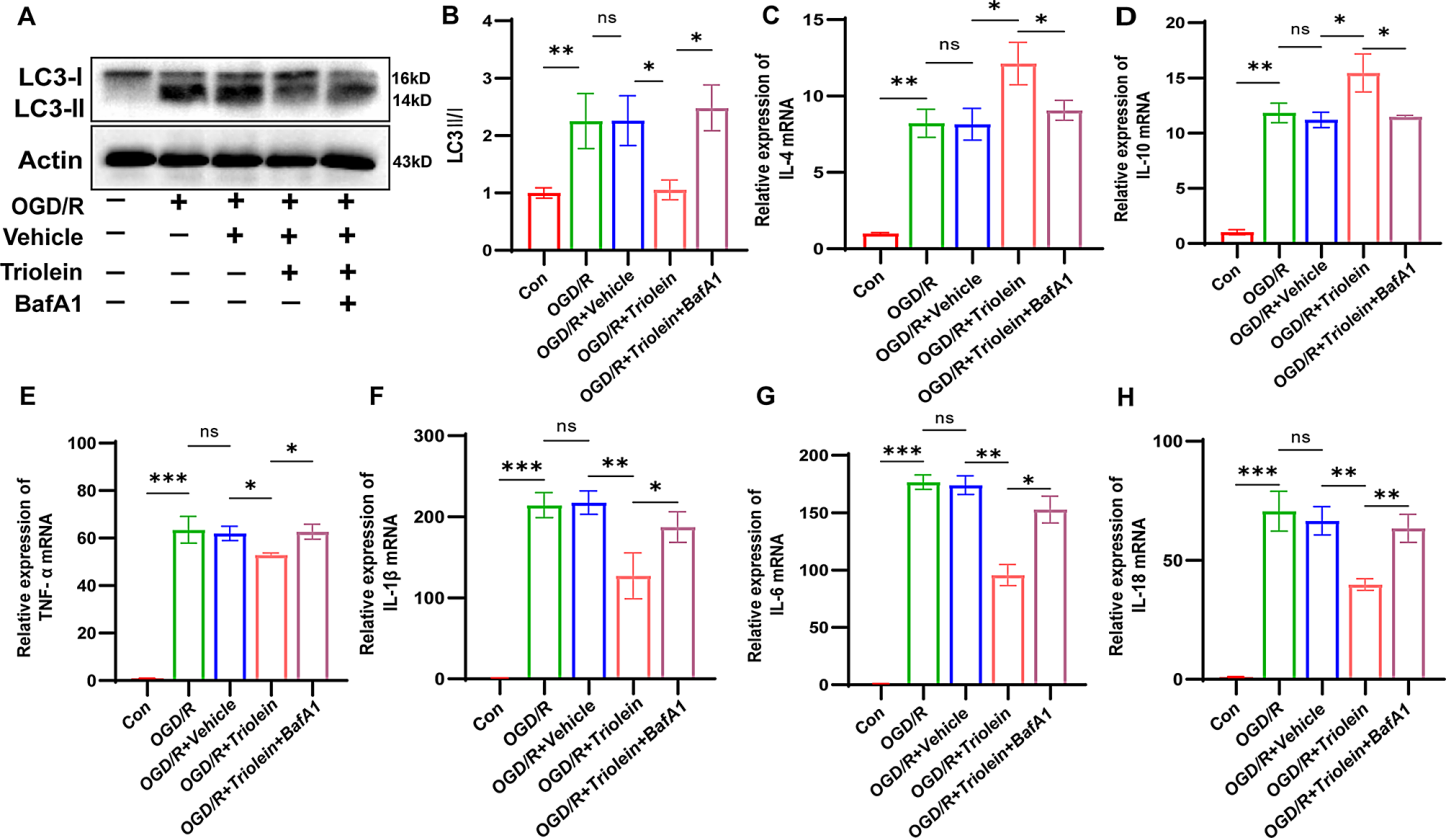
Triolein repressed OGD/R-induced inflammation in an autophagy-dependent manner. **A**–**B** The expression of LC3 and P62 was assessed by western blot in OGD/R induced BV2 treated with Triolein after BafA1 treatment. Quantitative results of relative band density are normalized to Actin. **C**–**H** The expression of IL-4, IL-10, TNF-α, IL-1β, IL-6, and IL-18 mRNAs in OGD/R induced BV2 after BafA1 was measured using RT-qPCR. GAPDH was used as the control. *n* = 3 per group. *p < 0.05, **p < 0.01, ***p < 0.001 versus the specified group. In addition, there was no statistically significant difference between the OGD/R group and the OGD/R + Vehicle group, indicating that the drug solvent had no additional effect on the experimental results

The CCK8 assay confirmed the minimal impact of specified concentrations of BafA1 on the viability of BV2 cells **(Supplementary S4)**. Western blot results (Fig. 5A–B) revealed that BafA1 administration increased the LC3-II/I ratio compared to the OGD/R + Triolein group, indicating the successful blockade of autophagy in the BV2-induced OGD/R model. A subsequent RT-qPCR application revealed changes in mRNA expression levels of IL-4, IL-10, TNF-α, IL-1β, IL-6, and IL-18. IL-4 and IL-10 showed decreased mRNA expression after BafA1 administration, whereas TNF-α, IL-1β, IL-6, and IL-18 showed increased mRNA expression compared to the OGD/R + Triolein group (Fig. 5C–H). These findings collectively suggest that autophagy inhibition effectively reverses triolein-mediated suppression of inflammation. In summary, preliminary findings show that triolein exerts anti-inflammatory effects in OGD/R-induced BV2 cells through an autophagy-dependent mechanism.

### Triolein exhibits a neuroprotective role by inhibiting autophagy

**Fig. 6:**
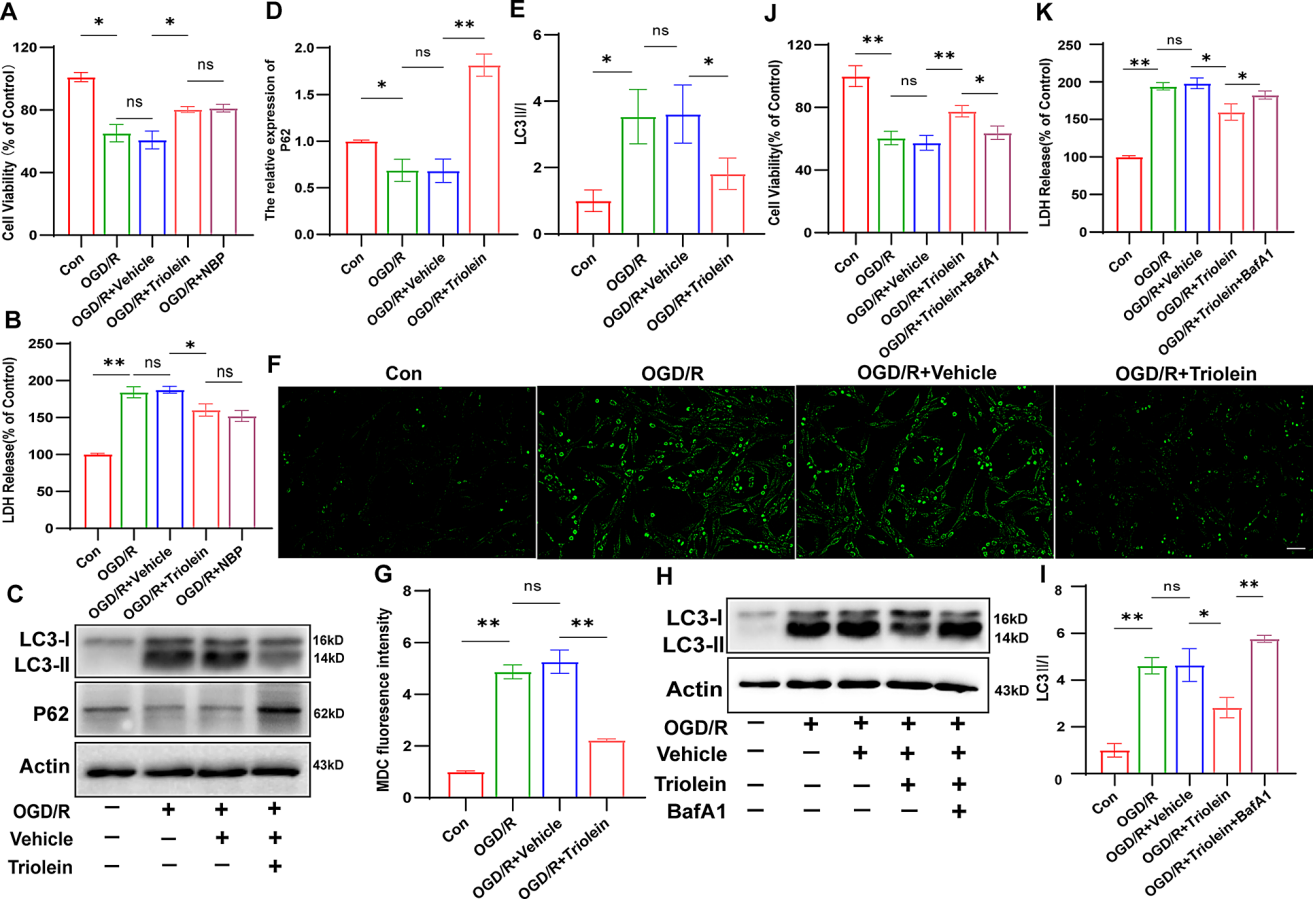
Triolein exhibits a neuroprotective role by inhibiting autophagy. **A** The neuroprotective effects of Triolein on OGD/R induced HT22 were evaluated using the CCK-8 assay. **B** The neuroprotective effects of Triolein on OGD/R induced HT22 were evaluated using the LDH assay. **C**–**E** The expression of LC3 and P62 in OGD/R induced HT22 treated with Triolein was assessed by western blot. Quantitative results of relative band density were normalized to Actin. **F**–**G** MDC labeled autophagic vesicles were used to detect the level of autophagy in OGD/R induced HT22 treated with Triolein (scale bar = 20 μm). **H**–**I** Western Blot analysis of LC3 after using BafA1 in OGD/R induced HT22. Quantitative results of relative band density are normalized to Actin. **J** The neuroprotective effects of Triolein on OGD/R induced HT22 after blocking autophagy were evaluated using the CCK-8 assay. **K** The neuroprotective effects of Triolein on OGD/R induced HT22 after blocking autophagy were evaluated using the LDH assay. *n* = 3 per group. *p < 0.05, **p < 0.01, ***p < 0.001 versus the specified group. In addition, there was no statistically significant difference between the OGD/R group and the OGD/R + Vehicle group, indicating that the drug solvent had no additional effect on the experimental results

Following OGD/R induction in BV2 cells, our investigation discovered the potential neuroprotective impact of triolein and its association with autophagy in HT22 cells. Similarly, we found that Triolein could protect HT22 cells from OGD/R by using NBP as a positive drug control through CCK8 and LDH release assays (Fig. 6A-B). Notably, quantitative analysis of P62 and LC3 proteins revealed that triolein effectively reduced the OGD/R-induced autophagic response in HT22 cells compared to the OGD/R + Vehicle group (Fig. 6C–E). Employing MDC to depict autophagic vacuoles further highlighted triolein’s ability to attenuate heightened autophagy levels elicited by OGD/R (Fig. 6F–G). The potential link between triolein’s neuroprotective effect and its autophagy inhibition in HT22 was explored. Effective autophagy inhibition through the autophagy antagonist BafA1 was found to reverse the augmented cell survival rate induced by triolein (Fig. 6H–K).

In summary, triolein can protect HT22 cells from OGD/R damage by inhibiting autophagy.

### Triolein decreases autophagy and inflammation and exerts neuroprotection through the AKT/mTOR signaling pathway

**Fig. 7:**
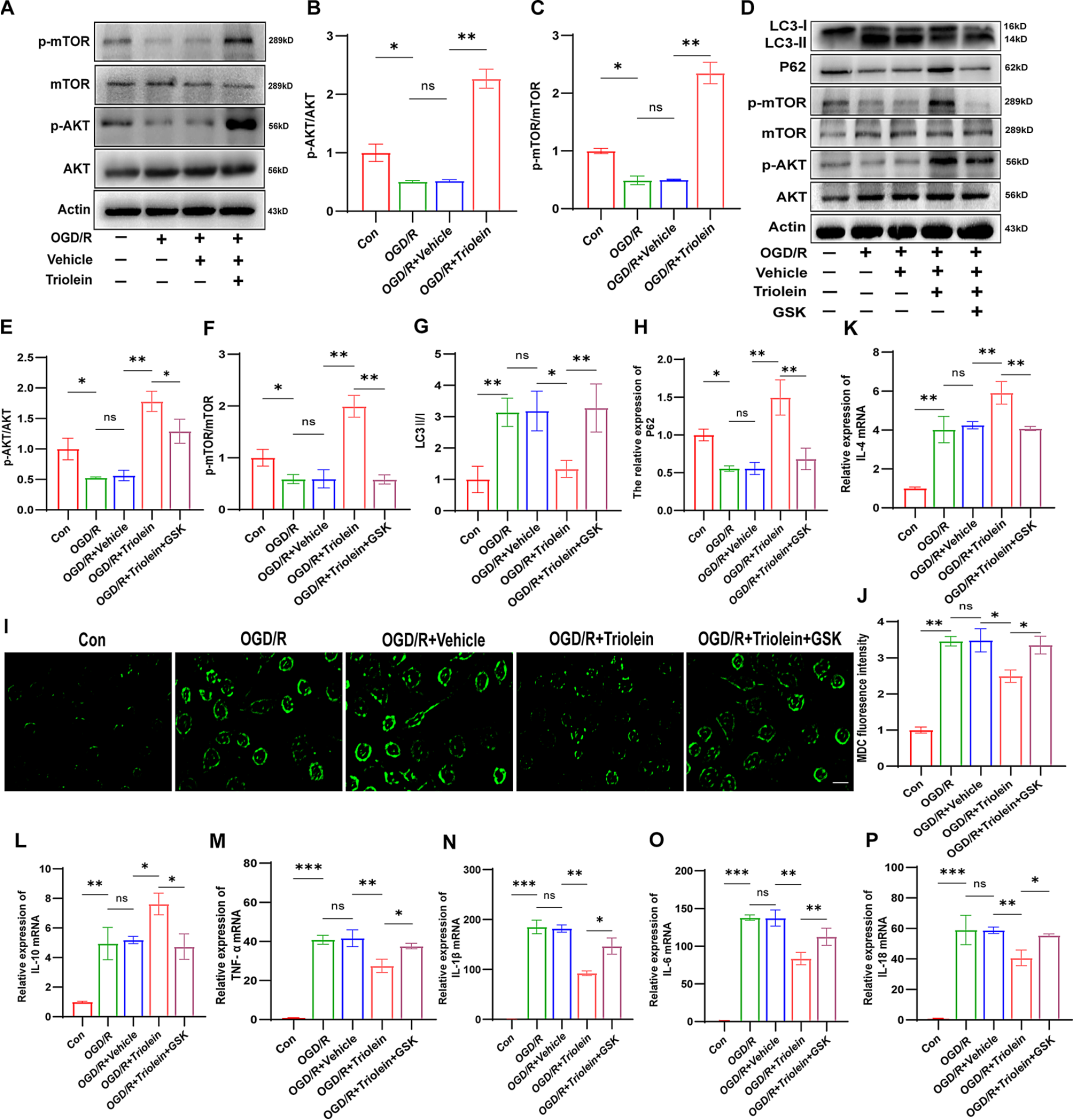
Triolein decreases autophagy and inflammation via AKT/mTOR signaling in OGD/R induced BV2. **A**–**H** The expression of AKT/mTOR signaling and autophagy related proteins in OGD/R induced BV2 after treatment with Triolein and GSK-690,693 was assessed by Western blotting assay. **I**–**J** MDC labeled autophagic vesicles were used to detect the level of autophagy after blocking the AKT/ mTOR pathway in OGD/R induced BV2. **K**–**P** The expression of the IL-4, IL-10, TNF-α, IL-1β, IL-6, and IL-18 mRNAs in OGD/R induced BV2 subjected to different treatments. GAPDH was used as the control. *n* = 3 per group. *p < 0.05, **p < 0.01, ***p < 0.001 versus the specified group. In addition, there was no statistically significant difference between the OGD/R group and the OGD/R + Vehicle group, indicating that the drug solvent had no additional effect on the experimental results

Our animal experiments revealed that triolein inhibited autophagy and the inflammatory response following stroke by activating the AKT/mTOR signaling pathway. We sought to validate these findings by conducting a comprehensive in vitro reexamination. As illustrated in Fig. 7A–C, the protein expression of p-AKT and p-mTOR in the OGD/R group was lower than that in the control group, signifying signaling pathway inhibition by OGD/R induction. Conversely, the OGD/R + Triolein group exhibited increased expression levels of p-AKT and p-mTOR compared to the OGD/R + Vehicle group, suggesting AKT/mTOR signaling pathway activation after triolein treatment. Subsequent experiments were conducted using AKT inhibitor GSK-690,693. The CCK8 assay confirmed that the GSK-690,693 concentration did not significantly affect the activity of BV2 and HT22 cells **(Supplementary S5)**. Utilizing the pathway inhibitor GSK-690,693 revealed reduced expression levels of p-AKT and p-mTOR proteins in the OGD/R + Triolein + GSK-690,693 group compared to the OGD/R + Triolein group, indicating successful suppression of AKT/mTOR signaling pathway activation by GSK-690,693 (Fig. 7D–F). These results revealed that total AKT and total mTOR protein expression levels exhibited no statistical differences among the groups.

Further examination was conducted to assess changes in inflammatory factors and autophagy after using GSK-690,693. The diminished expression of P62 and the elevated expression of LC3-II after inhibitor treatment compared to the OGD/R + Triolein group suggested that post-stroke autophagy inhibition by triolein was significantly attenuated following AKT/mTOR signaling pathway inhibition (Fig. 7G–H). In OGD/R-induced BV2 cells, inhibiting the AKT/mTOR signaling pathway also reversed the effect of triolein on OGD/R-induced excessive autophagy, as demonstrated by MDC-labeled autophagic vacuoles (Fig. 7I–J). Regarding inflammatory factors, the mRNA levels of IL-4 and IL-10 were significantly decreased, while those of TNF-α, IL-1β, IL-6, and IL-18 were significantly increased in the OGD/R + Triolein + GSK-690,693 group compared to the OGD/R + Triolein group. This suggests that inhibiting the AKT/mTOR signaling pathway partially counteracted triolein’s ability to inhibit post-stroke inflammation (Fig. 7K–P).

**Fig. 8:**
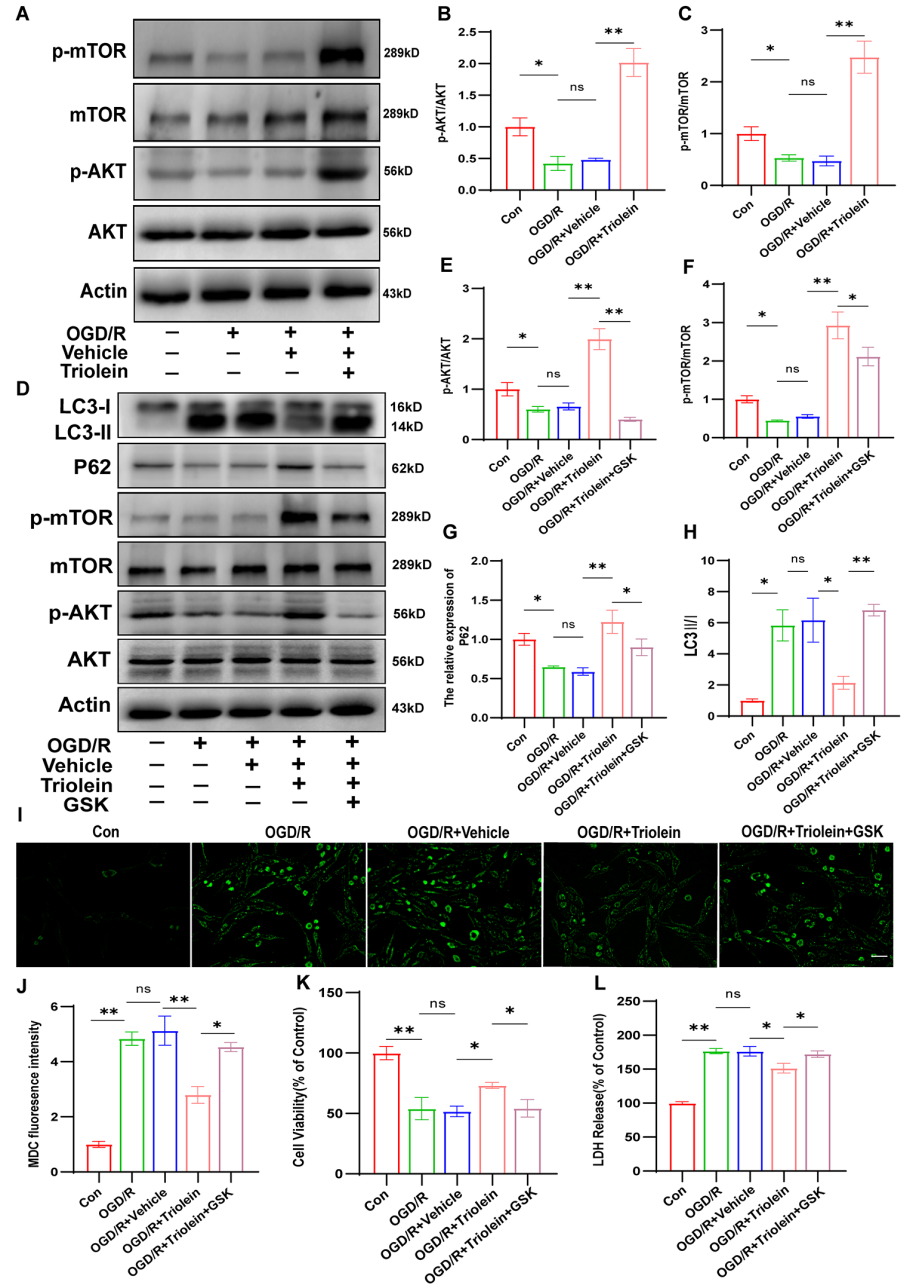
Triolein inhibits inflammation and autophagy via AKT/mTOR signaling in HT22-induced OGD/R in vitro. **A**–**H** The expression of AKT/mTOR signaling and autophagy related proteins in OGD/R induced HT22 after treatment with Triolein and GSK-690,693 was assessed by Western blotting. **I**–**J** MDC labeled autophagic vesicles were used to detect the level of autophagy after blocking the AKT/mTOR pathway in OGD/R induced HT22. **K** The neuroprotective effects of Triolein on OGD/R induced HT22 after blocking the AKT/mTOR pathway were evaluated using the CCK-8 assay. **L** The neuroprotective effects of Triolein on OGD/R induced HT22 after blocking the AKT/mTOR pathway were evaluated using the LDH assay. *n* = 3 per group. *p < 0.05, **p < 0.01, ***p < 0.001 versus the specified group. There was no statistically significant difference between the OGD/R group and the OGD/R + Vehicle group, indicating that the drug solvent had no additional effect on the experimental results

Collectively, these results indicate that the AKT/mTOR signaling pathway is implicated in triolein’s inhibition of post-stroke inflammation and autophagy. Furthermore, triolein’s neuroprotective function appears dependent on AKT/mTOR signaling pathway activation, findings substantiated in HT22 cells (Fig. 8). Similarly, triolein activated the AKT/mTOR signaling pathway in the HT22-induced OGD/R model (Fig. 8A–C). However, upon inhibiting this pathway (Fig. 8D–F), triolein’s protective effect, including autophagy regulation (Fig. 8G–J) of HT22 cells, was weakened or abolished. (Figs. 8K–L) and Diagram 1.

### Triolein exerts neuroprotection by AKT/mTOR signaling

**Diagram. 1:**
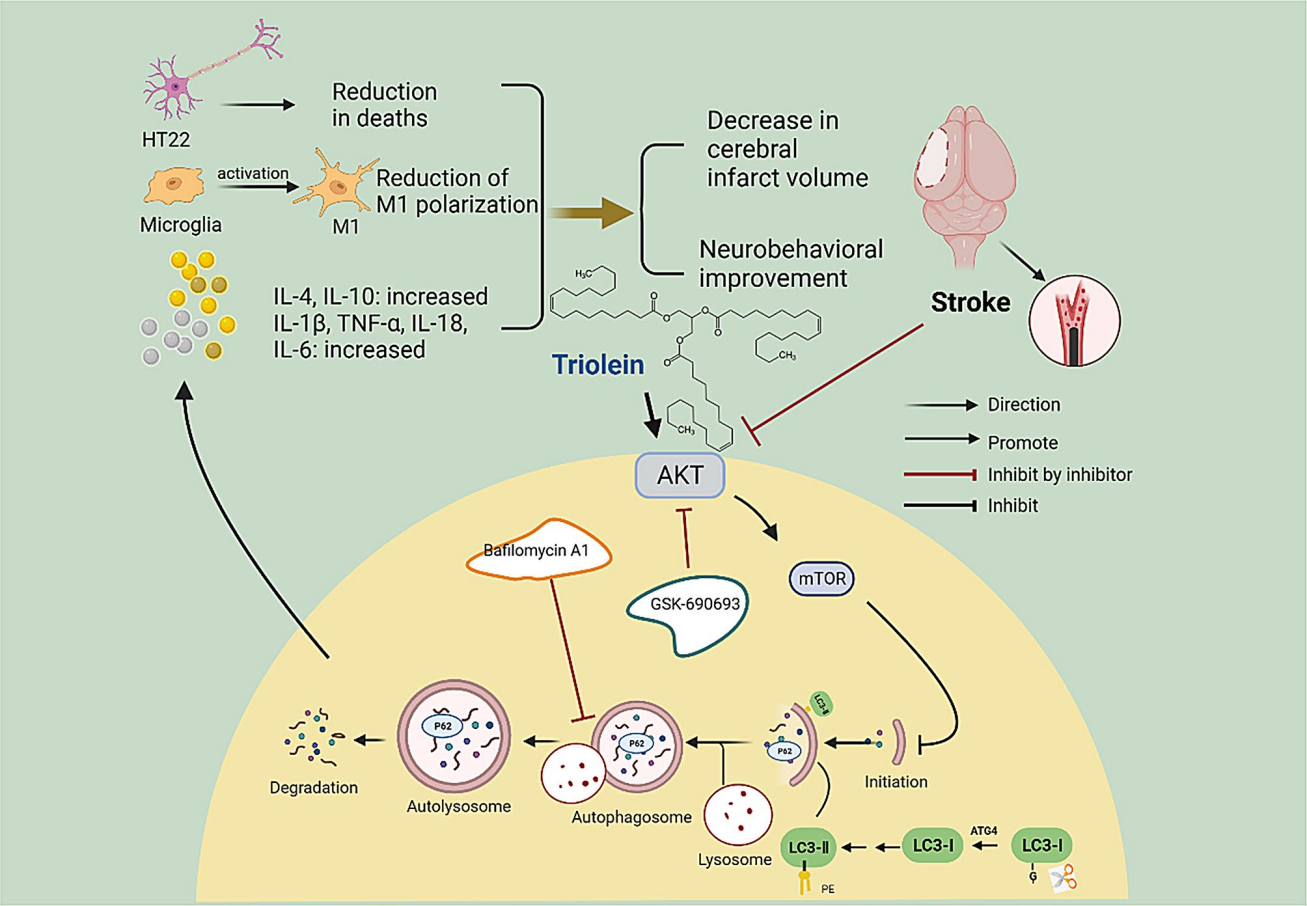
Schematic representation of the effect of Triolein on brain damage after ischemic stroke. Triolein, as demonstrated in a mouse model of middle cerebral artery occlusion (MCAO/R) through the Suture-Occluded Method, exhibited notable efficacy in reducing cerebral infarction size and facilitating the recovery of neurological function. Mechanistically, Triolein activated the AKT/mTOR signaling pathway, further inhibited the autophagy process, inhibited the release of inflammatory factors, decreased the polarization of M1 microglia, and increased the survival of HT22 cells. The observed effects were found to be partially counteracted by the administration of GSK-690693 and Bafilomycin A1. Created with BioRender.com

## Discussion

The presented research reveals, for the first time, the neuroprotective efficacy of triolein during the acute phase of IS. Our findings elucidate that triolein administration diminishes infarct volume, enhances neurological function, and mitigates the polarization of microglia in the ischemic brain, diverting microglia from the M1 phenotype associated with neurological injury and tissue destruction. Furthermore, our study revealed that triolein exerts an inhibitory effect on the inflammatory response and autophagy through AKT/mTOR signaling pathway activation, concurrently enhancing OGD/R-induced HT22 cell viability. Remarkably, in vitro studies demonstrated that triolein could reduce the OGD/R-induced inflammatory response in an autophagy-dependent manner Diagram 1.

The critical role of the inflammatory response in cerebral ischemia and reperfusion injuries has been extensively established in recent years (Iadecola et al. 2020). Most inflammatory cytokines are known to play harmful roles in IS (Zhu et al. 2022a, b). Our observations revealed a significant surge in pro-inflammatory cytokines, including IL-1β, IL-18, and IL-6, in the ischemic penumbra. Notably, triolein administration exerted a substantial influence, manifesting in a marked reduction in pro-inflammatory cytokines and a concomitant elevation in inflammation-suppressing cytokines, including IL-4 and IL-10, within the ischemic penumbra (Fig. 2A–F).

Microglia are the main immune cells in the brain and the first line of defense against brain injury. Microglia have long been categorized into two opposite functional states, pro-inflammatory M1 and anti-inflammatory M2, based on corresponding markers (Qin et al. 2019). Although a dichotomous classification may seem oversimplified, it is still widely used to assess the cellular inflammatory profile of microglia. The M1 phenotype is characterized by the production and release of pro-inflammatory mediators (TNF-α, Monocyte Chemotactic Protein-1 (MCP-1), and Inducible Nitric Oxide Synthase (iNOS), which accelerate cell death and exacerbate inflammation (Xian et al. 2022). Consequently, impeding the phenotypic transition of microglia toward the M1 phenotype emerges as a potential therapeutic strategy for mitigating IS-induced brain injury (Jin et al. 2023). Our findings revealed that MCAO/R induced the increased aggregation of microglia toward the ischemic penumbra and heightened the polarization of the M1 phenotype, as shown by immunofluorescence labeling of the microglia membrane surface marker Iba1 and M1 microglia membrane surface marker CD86. Notably, we found that triolein was able to reduce the aggregation of microglia and the polarization of the M1 phenotype (Fig. 2G); however, the mechanisms remain unclear and warrant further exploration.

Autophagy is a process in which eukaryotic cells utilize lysosomes to degrade their own cytoplasmic proteins and damaged organelles under the regulation of Atg (Debnath et al. 2023). Like inflammation, autophagy plays a double-edged role in cerebral ischemia-reperfusion injury. Autophagy inhibition was demonstrated to reduce cerebral infarct size and ameliorate neurological deficits in MCAO/R mice (Cao et al. 2023). In vitro experiments have also demonstrated that autophagy inhibition restores cell viability, reduces LDH levels, and has a protective effect against OGD/R-induced HT22 cell damage (Wang et al. 2022a, b; Zhang et al. 2023a, b). Furthermore, it has been shown that preventing excessive autophagy contributes to thalamic angiogenesis and influences functional recovery after stroke (Xiao et al. 2022). LC3-II is a signature protein of autophagy. During autophagy, LC3-I connects to phosphatidylethanolamine (PE) facilitated by the Atg5-Atg12-Atg16 complex to form LC3-II, which localizes to the outer membrane of the autophagosome and initiates the autophagy process. P62 is an autophagic degradation substrate crucial for recognizing and encapsulating degradation substrates. Therefore, LC3 and P62 have been widely studied as signature proteins of autophagy (Kim and Lee 2014). Likewise, our findings demonstrated that autophagy plays a deleterious effect on IS and that triolein-mediated autophagy repression correlates with reduced infarct volume and improved neurological function (Figs. 1 and 2H). In vitro, in OGD/R-induced HT22 cells, our study validated that triolein inhibits excessive autophagy post-stroke, which restores attenuated cell viability and reduces LDH levels, thereby exerting a protective effect against OGD/R injury (Fig. 6). Triolein treatment reversed MCAO/R- or OGD/R-induced changes in the ratio of LC3-II/I expression and P62 levels. Furthermore, MDC staining showed that OGD/R injury exacerbated autophagy, which was inhibited by triolein. To determine autophagy levels, Western blotting and MDC staining were employed. However, since these methods represent static measurements of autophagy levels, capturing the changes in autophagy dynamics is difficult. Therefore, our future research will focus on utilizing more advanced or comprehensive autophagy measurement methods, including fusion protein labeling and electron microscopy, which can observe autophagy more precisely and dynamically.

Inflammation and autophagy mutually interact and regulate each other in neurological diseases like IS. Autophagy and inflammation have both antagonistic and synergistic functions (Yang et al. 2015; Shi et al. 2018). Regarding the relationship between autophagy and inflammation, Nakahira et al. (Nakahira et al. 2011). found that depleting autophagy proteins LC3B and Beclin-1 enhanced lipopolysaccharide-induced secretion of IL-1β and IL-18 in LC3-II-deficient mice. Similar results were obtained in our study, in which the ability of triolein to inhibit the expression of pro-inflammatory cytokines (IL-1β, TNF-α, IL-6, and IL-18) and enhance the expression of anti-inflammatory cytokines (IL-4 and IL-10) was significantly reduced upon autophagy blockage in the BV2-induced OGD/R model (Fig. 5). These findings infer that the anti-inflammatory effects exerted by triolein in ischemic injury partly depend on autophagy regulation. The intercommunication between autophagy and inflammation is complex and warrants further exploration. In the present study, autophagy inhibitor BafA1 was used to inhibit autophagy in vitro before detecting inflammation. Future studies could utilize genetically engineered mice to silence key autophagy genes to corroborate our experimental findings.

Our study further explored the potential molecular mechanisms by which triolein regulates autophagy and inflammation after stroke. To date, the AKT/mTOR signaling pathway plays an essential role in regulating inflammation and autophagy, especially in autophagy regulation (Fang et al. 2021). When this signaling pathway is triggered, autophagy is suppressed (Huang et al. 2022). Previous studies have shown that AKT/mTOR activation can reduce IS-induced brain injury (Zheng et al. 2021; Li et al. 2023). To clarify whether the AKT/mTOR signaling pathway participates in the neuroprotective function of triolein in IS, this study selected GSK-690,693 to inhibit AKT function, followed by detecting changes in the expression of Atg and inflammatory factors and assessing OGD/R-induced HT22 injury. Western blot results showed that p-AKT and p-mTOR protein expression was decreased in the MCAO/R + Vehicle group, whereas triolein increased this expression 3 days post-IS. In both in vivo and in vitro experiments, inhibiting the AKT/mTOR signaling pathway with GSK-690,693 significantly reversed the inhibitory effect of triolein on inflammation (Figs. 3 and 7) and increased HT22 cell viability (Fig. 8). Overall, these results indicate the involvement of the AKT/mTOR signaling pathway in the crucial function of triolein in exerting anti-inflammatory effects and inhibitory effects on autophagy in IS-induced brain injury. However, we could not completely elucidate whether triolein regulates other inflammation- or autophagy-related signaling pathways. Moreover, how triolein regulates the AKT/mTOR signaling pathway remains unclear and warrants exploration in future studies. Currently, diverse perspectives exist regarding the impact of ischemic stroke on the AKT/mTOR signaling pathway. Some propose that MCAO/R triggers AKT/mTOR signaling pathway activation (Liu et al. 2018). This contradictory view may be attributed to multiple factors. First, the type of IS model plays a pivotal role. Variations in modeling techniques or ischemic durations can yield distinct degrees of pathophysiological alterations, thereby influencing the activity of the AKT/mTOR signaling pathway. Moreover, different analytical methods, including Western blotting or immunohistochemistry, may lead to differences in sensitivity to different aspects of signaling pathway activity. Choosing an appropriate analytical method is essential to achieve consistent results. Finally, IS is a complex and dynamic disease process, and different time points may exhibit different signaling pathway activity. Collectively, these factors may all contribute to the observed inconsistency in signaling pathway activity. Therefore, future investigations should further explore these discrepancies to attain a more comprehensive and profound understanding of the mechanistic intricacies of the AKT/mTOR signaling pathway in IS.

The present investigation serves as an initial exploration of triolein, revealing for the first time its novel protective role in both in vivo and in vitro experiments in IS and delineating its possible mechanism. However, certain limitations and expectations must be acknowledged. Firstly, IS predominantly affects the elderly population, whereas our in vitro experimental mice were young. Additionally, further studies of triolein’s complex mechanisms are anticipated to advance the development of neuroprotective drugs to make them safer and more effective. Comprehensive studies, including clinical trials, are imperative to confirm the safety and efficacy of triolein as a prospective adjuvant therapy to conventional chemotherapy regimens. The triolein dose and timing of administration used in this study were based on the results of the pilot experiment, but this may not be the optimal protocol. Different doses or different time points of administration may produce different therapeutic effects. In addition, the use of a single dose limits the opportunity to understand the drug dose-dependent relationship. In the future, the pharmacological effects of multiple drug doses should be explored.

The prospect of triolein as an IS drug appears promising, especially given its demonstrated ability to regulate key pathways involved in neuroprotection. Its dual role in modulating both autophagy and inflammation—critical processes involved in the pathophysiology of IS—positions triolein as a potential multifunctional therapeutic agent. Moving forward, it will be crucial to assess not only the therapeutic efficacy of triolein in more clinically relevant models but also its safety profile in long-term treatments.

## Conclusion

In conclusion, our study elucidates, for the first time, the neuroprotective role of triolein in IS, achieved through autophagy-dependent inhibition of the post-IS inflammatory response. Furthermore, the neuroprotective effects of triolein are intricately linked to the AKT/mTOR signaling pathway. Consequently, this research signifies triolein as a promising new therapeutic candidate for IS.

## Electronic supplementary material

Below is the link to the electronic supplementary material.

Supplementary Material 1

Supplementary Material 2

Supplementary Material 3

Supplementary Material 4

Supplementary Material 5

Supplementary Material 6

Supplementary Material 7

## Data Availability

No datasets were generated or analysed during the current study.

## Abbreviations

OGD/R: Oxygen-Glucose Deprivation/Reoxygenation
MCAO/R: Middle Cerebral Artery Occlusion
RT-qPCR: Real-Time Quantitative PCR
TTC: 2,3,5-Triphenyl Tetrazolium Chloride
MDC: Monodansyl Cadaverine
WB: Western Blot
IS: Ischemic Stroke
MMPs: Matrix Metalloproteinases
TNF-α: Tumor Necrosis Factor-alpha
IL-1β: Interleukin-1β
IL-6: Interleukin-6
ROS: Reactive Oxygen Species
NLRP3: NOD-Like Receptor Thermal Protein Domain Associated Protein 3
LC3: Light Chain 3
MCP-1: Chemotactic Protein-1
PE: Hosphatidylethanolamine
