# Supplementary figures and images for: Triolein alleviates ischemic stroke brain injury by regulating autophagy and inflammation through the AKT/mTOR signaling pathway
Source: Mol Med. 2024 Dec 6;30:242. doi: 10.1186/s10020-024-00995-5 (PMC11622655; doi:10.1186/s10020-024-00995-5)

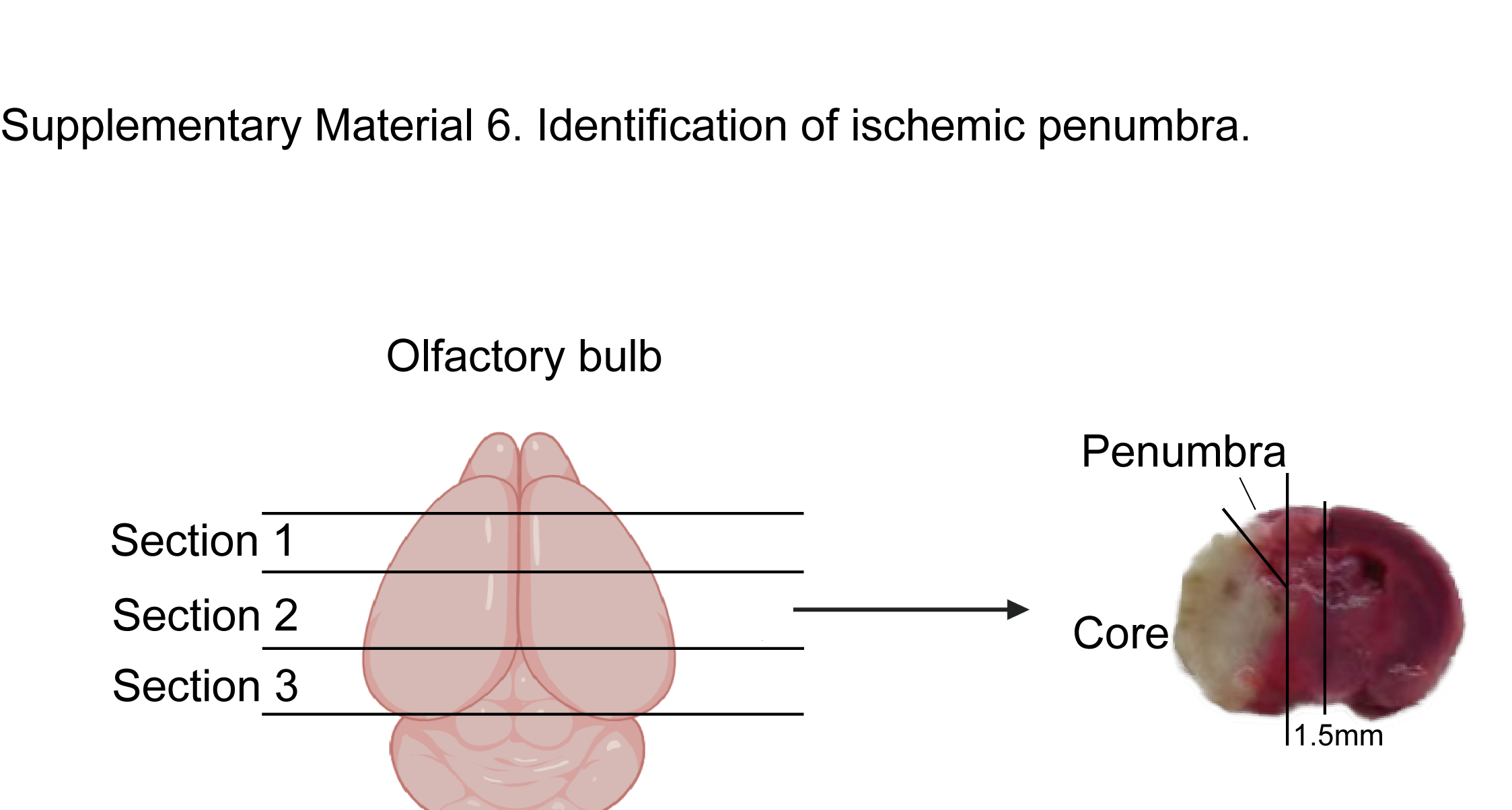

Supplement: Supplementary file 7 — Supplementary Material 7 [file 10020_2024_995_MOESM7_ESM.png]
